# Targeting the Zika virus envelope domains I and III as a recombinant vaccine protects mice from lethal challenge

**DOI:** 10.1038/s41541-026-01442-8

**Published:** 2026-04-14

**Authors:** Vincent Dussupt, Jaime L. Jensen, Angélica Peña Rosado, Marissa Donofrio, Jill Pflugheber, Letzibeth Mendez-Rivera, Rajeshwer S. Sankhala, Wei-Hung Chen, Bonnie M. Slike, Annika Schmid, Ursula Tran, Lily Metzger, Caroline E. Peterson, Amelia K. Pinto, Sandhya Vasan, Natalie D. Collins, Aaron Farmer, Nelson L. Michael, M. Gordon Joyce, James D. Brien, Shelly J. Krebs

**Affiliations:** 1 Viral Diseases Program, Walter Reed Army Institute of Research, Silver Spring, MD USA; 2 U.S. Military HIV Research Program, Walter Reed Army Institute of Research, Silver Spring, MD USA; 3 Henry M. Jackson Foundation for the Advancement of Military Medicine, Bethesda, MD USA; 4 Center for Infectious Disease Research, Walter Reed Army Institute of Research, Silver Spring, MD USA; 5 Microbiology, Immunology, and Molecular Genetics, University of Kentucky, Lexington, KY USA

**Keywords:** Biotechnology, Immunology, Microbiology

## Abstract

Zika virus (ZIKV) vaccine candidates developed through Phase I clinical trials are based on the full-length envelope glycoprotein (E), which presents both desirable and undesirable antigenic determinants. Among the latter, the conserved fusion loop epitope (FLE) within domain II is a major target for flavivirus cross-reactive and poorly neutralizing responses. To eliminate unwanted FLE targeting, we redesigned ZIKV E using a reverse vaccinology approach, excising domain II and allowing domains I and III (DI-DIII) to fold into an independent subunit harboring key neutralizing epitopes. *Ifnar1*^*-/-*^ mice vaccinated with ZIKV DI-DIII elicited high ZIKV neutralizing antibodies and were protected from weight loss and death. In addition, sera from DI-DIII vaccinated mice demonstrated a reduced capacity to enhance DENV 1-4 infection in vitro, compared to mice vaccinated with full-length E. This study identifies DI-DIII as a promising immunogen, focusing antibody responses to protective epitopes on ZIKV and minimizing the elicitation of unwanted responses.

## Introduction

Zika virus (ZIKV) is a mosquito-borne, positive-sense RNA flavivirus of the *Orthoflavivirus* genus discovered in non-human primates in the Zika Forest, Uganda in 1947 and first described in 1952^1^. ZIKV raised clinical concerns during the 2007 Yap Island outbreak^2^, followed by the 2013 French Polynesia outbreak where links to a rare neurological disorder, Guillain-Barré syndrome, were established^3^. Shortly thereafter, ZIKV was discovered in Brazil and started to spread throughout the Americas^4^ resulting in the largest ZIKV epidemic to date. The Central and South American outbreak of 2015–2016 was declared a public health emergency of international concern by WHO, following numerous cases of congenital neurodevelopmental defects, collectively known as congenital Zika syndrome, a consequence of vertical transmission of ZIKV during pregnancy. Another unique feature of ZIKV discovered in this large outbreak was the evidence for sexual transmission, likely associated with viral persistence in the male reproductive system were ZIKV can be detected for months after infection (reviewed in ref. ^5^). Although cases of ZIKV have declined globally since 2017, there is continued low level ZIKV transmission throughout the globe; ZIKV is poised to re-emerge and cause significant disease in parts of the world where its mosquito vector is present and natural immunity wanes. In 2025, more than 21,000 cases of ZIKV were reported in Brazil [https://www.paho.org/en/arbo-portal/zika-data-and-analysis/zika-analysis-country], with new cases seen in Yemen and Burkina Faso. Development of medical countermeasures, such as vaccines or therapeutic monoclonal antibodies (mAbs), are needed to protect or treat at-risk populations who live in or travel to ZIKV-endemic regions.

The major target of neutralizing and protective antibodies is the ZIKV envelope glycoprotein (E), the only accessible protein at the surface of mature flaviviruses^6^. Each E monomer is composed of three domains, DI, DII and DIII, with DI linking the putative host cell receptor binding domain DIII to DII, the dimerization domain that also harbors the fusion loop at its distal end^7^. Following the 2015-2016 ZIKV outbreak, several vaccine platforms quickly entered Phase I clinical trials^8^. Those platforms included purified inactivated virus^9,10^, DNA-based^11^, mRNA-based^12^ and recombinant vector-based^13^ platforms, which all utilized the full-length ZIKV E glycoprotein. Whereas most of these vaccines were highly immunogenic and safe, the advancement into Phase III efficacy trials was halted due to decreasing ZIKV transmission, and the lack of a human challenge model at the time^14^. Therefore, there are currently no licensed ZIKV vaccines available to date.

During that time, the Walter Reed Army Institute of Research (WRAIR) developed and evaluated a ZIKV purified inactivated virus vaccine candidate (ZPIV) in non-human primates and in a series of Phase I clinical trials^9,15–17^. Potent ZIKV neutralizing antibodies were elicited and correlated with protection from infection in non-human primates^16,17^. From these Phase I clinical samples, we identified a class of potent ZIKV-dengue virus (DENV) cross-neutralizing monoclonal antibodies protective against both ZIKV and DENV-2 viral challenges^18^. The prototypical mAb, MZ4, engages a conformational epitope on the ZIKV virions primarily through the DI/DIII linker with secondary contacts in DI and DIII^18^. More recently, structural characterization of A9E^19^, another potent ZIKV neutralizing antibody, identified an epitope similar to that of MZ4, underlining DI-DIII as a functional subunit and a site of vulnerability on the ZIKV E glycoprotein. Previous work by others have also identified the lateral ridge (LR) epitope in DIII as an epitope of protective value^20–25^, making DI-DIII an attractive target for immunogen design. We hypothesized that a novel ZIKV immunogen based on the DI-DIII subunit would display several epitopes of ZIKV protective antibodies and may benefit from the absence of DII and its highly conserved fusion loop epitope (FLE). FLE is well-known for being a target for cross-reactive and poorly neutralizing antibody responses that have also been associated with antibody-dependent enhancement (ADE) of infection^26^. ADE is suspected to occur during heterotypic secondary DENV infection, where non-or sub-neutralizing pre-existing IgG may opsonize viral particles and facilitate intake into cells harboring Fcγ receptors at their surface, thereby increasing viral replication and disease. Given the close relatedness of ZIKV and DENV, evidence from clinical and epidemiological studies, as well as B cell repertoire analyses, point to complex interactions between ZIKV and DENV, encompassing both protective and enhancing antibody responses^27^.

Here, we describe reverse engineering efforts to rationally design a recombinant ZIKV DI-DIII subunit immunogen devoid of the FLE-containing DII. The recombinant ZIKV DI-DIII binds with high affinity to potently neutralizing antibodies that target the DI/DIII linker and the DIII LR epitope. We investigated the use of ZIKV DI-DIII as an E subunit immunogen and demonstrated the elicitation of neutralizing antibodies and protection against a ZIKV lethal challenge mouse model. Mice immunized with the ZIKV DI-DIII immunogen exhibited potent ZIKV neutralizing antibodies with reduced enhancement of DENV-2 infection in vitro, as compared to full-length E. Our proof-of-concept study provides a basis for the development of the ZIKV DI-DIII recombinant immunogen as a vaccination approach aimed at eliciting protective immunity towards neutralization epitopes on ZIKV virions.

## Results

### Engineering of the ZIKV E DI-DIII subunit

**Fig. 1:**
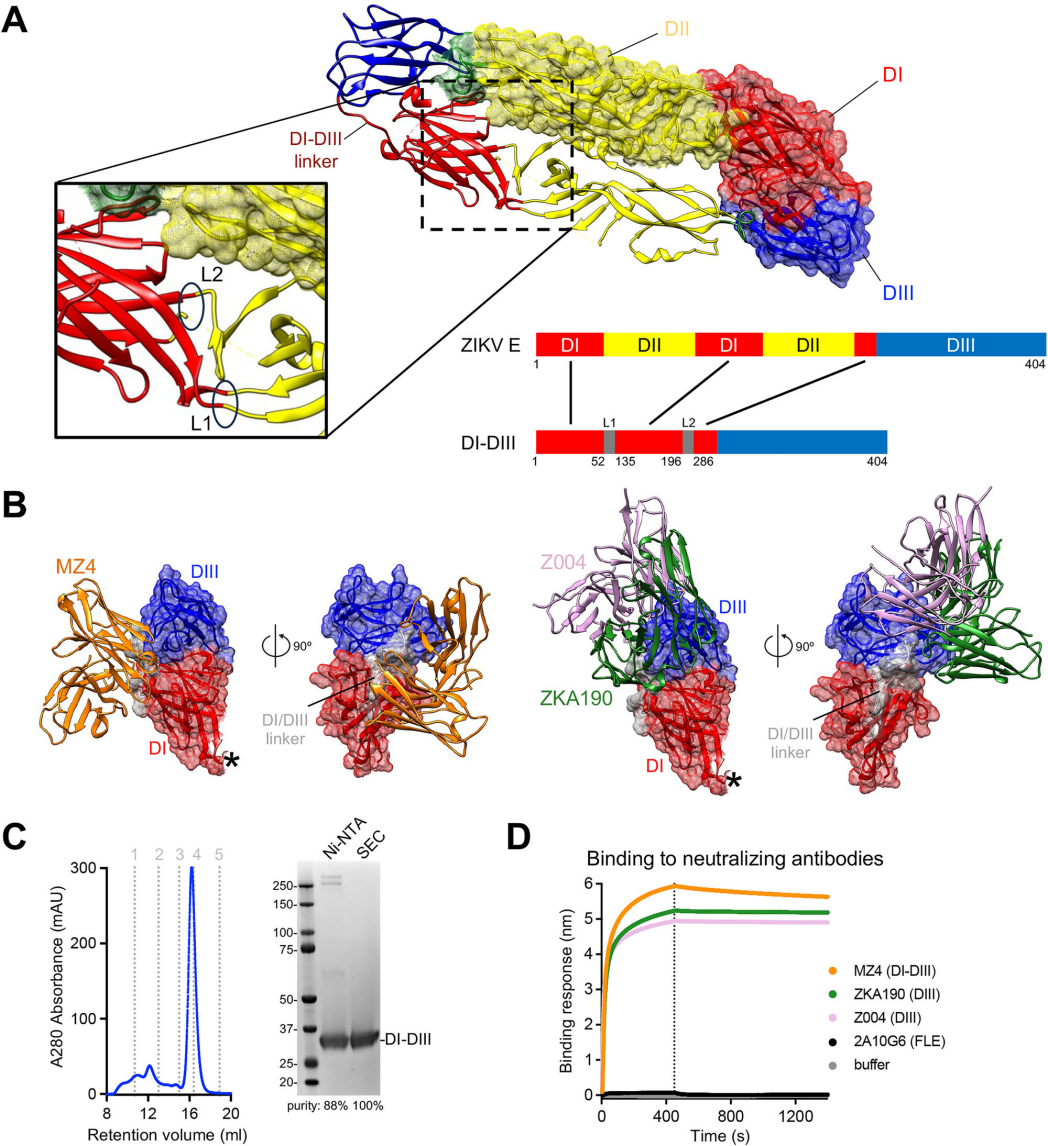
Design, expression and characterization of a ZIKV E DI-DIII subunit. **A** Top, Representation of the ZIKV E dimer (PDB 5LBV) with one E monomer represented as ribbon and the other depicted as ribbon under a transparent mesh surface. Domains I, II and III are colored in red, yellow and blue, respectively, while the fusion loop in DII is highlighted in green. Insert indicates a close-up on the DI/DIII interface where glycine linkers (L1 and L2) were engineered. Bottom, design of a DI-DIII subunit devoid of DII, with glycine linkers (gray) connecting the DI polypeptides in absence of DII. Amino acid positions are indicated using ZIKV E numbering. **B** Depiction of DI-DIII, as ribbon under a transparent mesh surface, along with epitopes of known potent ZIKV neutralizing antibodies (as Fv) that recognize the DI/DIII interface (MZ4, orange, this work, left panel) or DIII (Z004, pink, PDB 6UTA; ZKA190, green, PDB 5Y0A, right panel), represented as ribbon. DI and DIII are colored in red and blue, respectively, with the DI/DIII linker in light gray. A black asterisk indicates the original position of DII in the E monomer. **C** Purification of ZIKV DI-DIII expressed in Drosophila S2 cells. Left, Size exclusion chromatography profile of ZIKV DI-DIII after Ni-NTA affinity purification. Gray dotted lines indicates molecular weight markers, 1: 670 kDa, 2: 158 kDa, 3: 44 kDa, 4: 17 kDa and 5: 1.3 kDa. Right, Coomassie stain after affinity (Ni-NTA) and size exclusion (SEC) chromatography. **D** Binding of control monoclonal antibodies to the ZIKV DI-DIII as assessed by BLI.

Building on our previous findings^18^ and collective knowledge of monoclonal antibodies targeting ZIKV protective epitopes^28^, we set out to construct a protein immunogen displaying ZIKV DI-DIII domains. To that end, we replaced the entire DII domain of ZIKV E, consisting of four polypeptide connections to DI, with two short glycine linkers to allow DI to fold into its native conformation in absence of DII (Fig. 1A). This redesigned immunogen allows for the presentation of key flavivirus epitopes targeted by potent neutralizing protective antibodies, such as the DI/DIII interface (MZ4^18^) and the DIII lateral ridge (LR) (Z004^21^, ZKA190^22^) (Fig. 1B). The resulting ZIKV DI-DIII protein was expressed from insect cells and purified to homogeneity by affinity and size exclusion chromatography, where it eluted as a single peak with a retention volume consistent with the molecular weight of a monomer (29 kDa) (Fig. 1C). To evaluate the folding and antigenicity of the purified recombinant ZIKV DI-DIII, we performed biolayer interferometry assays with a set of ZIKV mAbs. The FLE-directed and pan-flavivirus cross-reactive mAb 2A10G6^29^ failed to interact, as expected, while neutralizing antibodies ZKA190^22^ and Z004^21^ that recognize DIII, and MZ4^18^ that recognizes the DI/DIII linker, all bound robustly to ZIKV DI-DIII, indicating that those important neutralization epitopes were conserved within the redesigned immunogen (Fig. 1D).

### Crystal structure of ZIKV DI-DIII in complex with the neutralizing antibody MZ4

**Fig. 2:**
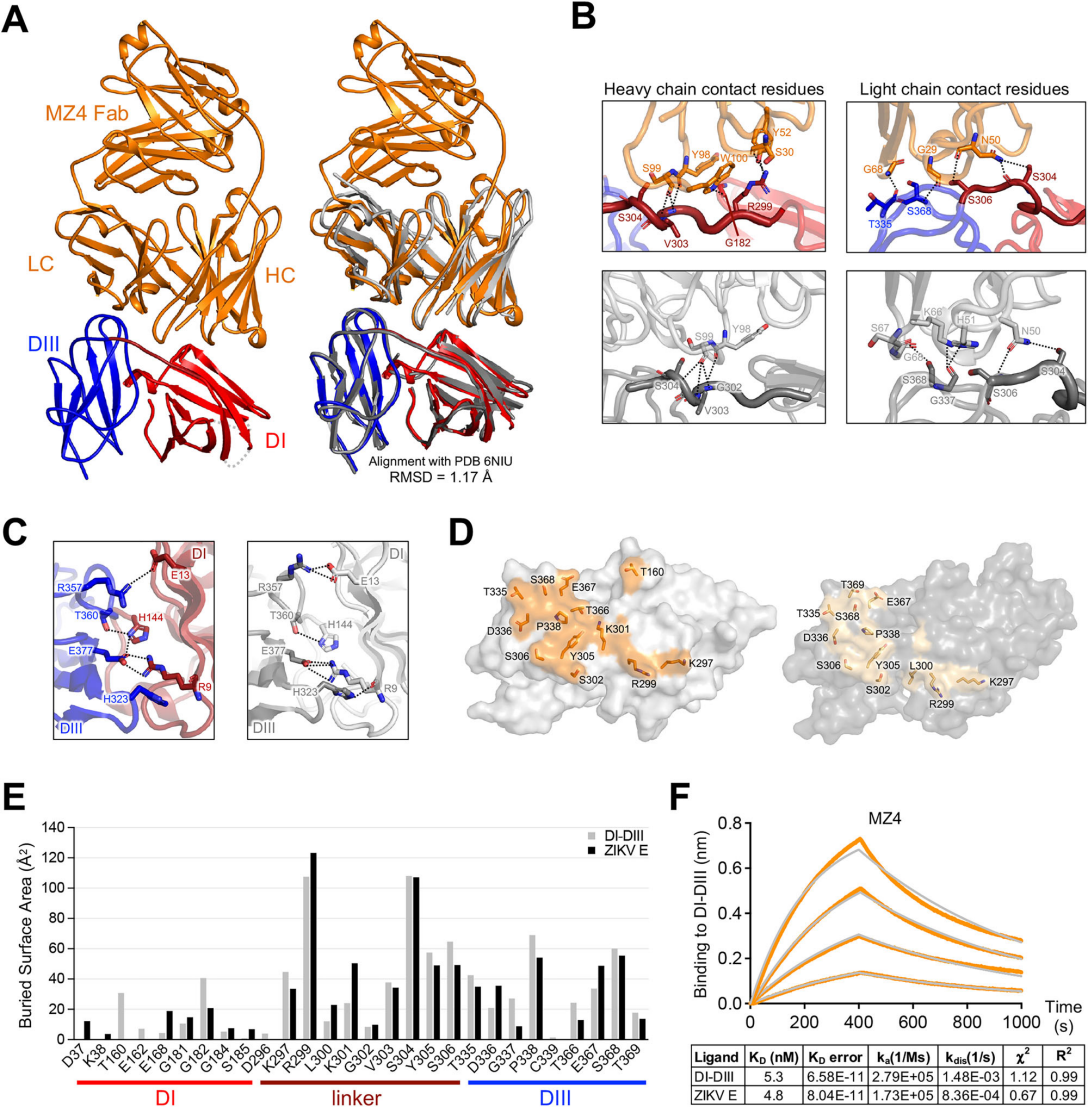
The recombinant DI-DIII subunit retains its native conformation and interacts with MZ4 in a similar fashion to the full-length E protein. **A** (Left) Crystal structure of the ZIKV DI-DIII–MZ4 complex. DI-DIII is represented in red and blue, respectively, while MZ4 Fab is in orange. (Right) The ZIKV DI-DIII–MZ4 complex, superimposed with the DI-DIII from ZIKV full-length E (ZIKV E) and MZ4 complex (gray; PDB 6NIU). DI-DIII from the two structures aligned across 191 C*α* atoms with a root mean square deviation (RMSD) of 1.17 Å. **B** Heavy and light chain contact residues between DI-DIII–MZ4 (top panels; colored as in A) and ZIKV E–MZ4 (bottom panels; gray). **C** Contact residues at the DI/DIII interface, colored as in panels **A**–**B**. **D** MZ4 footprint on the DI-DIII subunits of the engineered DI-DIII (left) and native ZIKV E (right). Only residues with buried surface area (BSA) > 20 Å^2^ are highlighted. **E** Differences in MZ4 interacting residues between the DI-DIII (this work) and ZIKV E (PDB 6NIU) structures across DI, DIII, and the DI-DIII linker. Plotted are BSA values of MZ4 contact residues in DI-DIII for the two structures, with values from the DI-DIII and ZIKV E structures in black and gray, respectively. **F** Left, Binding kinetics of MZ4 to ZIKV DI-DIII. Affinity constants were calculated from binding curves obtained from 4 serial dilutions of Fab and fitted (gray curves) using a 1:1 binding model. Right, Summary of BLI binding kinetic constants and fit parameters obtained with MZ4 Fab and either DI-DIII (this work) or ZIKV E^14^.

To confirm that the engineered ZIKV DI-DIII subunit retains its native conformation, we determined the crystal structure of ZIKV DI-DIII in complex with MZ4 Fab to a resolution of 2.85 Å (Fig. 2A; Supplementary Table 1). Using pairwise structure alignment with the previously reported structure of MZ4 in complex with full-length E resolved to 4.3 Å (PDB ID 6NIU)^18^, we observed that the isolated DI-DIII adopts a conformation nearly identical to its original fold within the full-length E, as determined by a root mean square deviation (R.M.S.D.) value of 1.17 Å across 191 C$\alpha$ atoms. The DI-DIII-MZ4 structure features 5 additional hydrogen bonds between the DI-DIII and the MZ4 heavy chain, as compared to the full-length E-MZ4 structure (Fig. 2B, left). Conversely, hydrogen bonds between the MZ4 light chain residues H51 and K66 and E residue G337 are not observed in the DI-DIII-MZ4 structure (Fig. 2B, right). The invariant residues (Fig. S1A) that form salt bridges and hydrogen bonds across the DI/DIII interface are maintained in the DI-DIII structure (Fig. 2C). We computed the buried surface area (BSA) for each contact residue (Fig. 2D–E); the DI-DIII-MZ4 shows a total BSA of 869 Å^2^, slightly larger than the E-MZ4 interface, with a total BSA of 833 Å^2^. The overall subtle differences between the two structures are more likely due to the improved data quality and resolution seen in the DI-DIII-MZ4 complex structure (Fig. S1B–C). As an example, we were able to resolve the NAG group of a glycan at N154 in the DI-DIII structure, which is accommodated by a shift of the loop between V153 and T160 by ~4 Å toward DIII, as compared to the MZ4-E complex structure (PDB ID: 6NIU) (Fig. S1D). The heavy and light chains of MZ4 Fab retained nearly identical conformations in the bound complex with either DI-DIII alone or with full-length E, with R.M.S.D. of 0.66 Å and 0.63 Å between the heavy chains and the light chains, respectively. In addition, comparing the binding kinetics of MZ4 Fab to the ZIKV DI-DIII subunit to binding kinetics of the MZ4 Fab to full-length E, revealed nearly identical affinity constants (K_D_) (Fig. 2F). Combined, these data suggest that the engineered ZIKV DI-DIII immunogen retains its native confirmation.

### In vivo protection study in mice

**Fig. 3:**
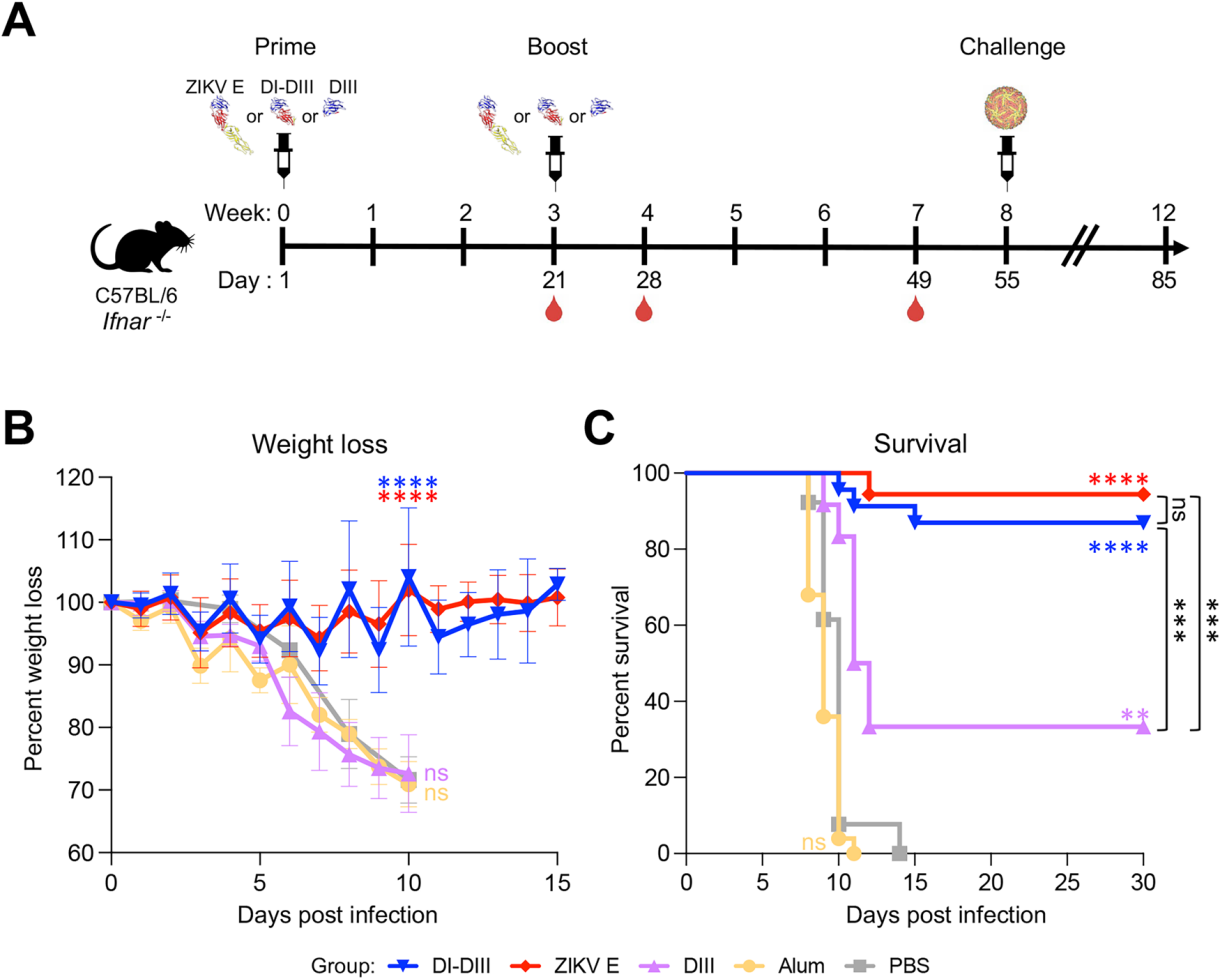
Immunization with ZIKV DI-DIII protects mice from lethal challenge. **A** C57BL/6 *Ifnar* ^*-/-*^ mice were immunized twice with 5 µg of the indicated immunogen in alhydrogel + CpG2395 (Alum) and challenged at day 55 with ZIKV (1 × 10^5^ FFU). **B** Weight curves of immunized animals during the first 15 days following ZIKV challenge. Data is an aggregate of 3 independent studies where *n* = 24 Alum, *n* = 15 DIII, *N* = 23 DI-DIII, and *n* = 18 ZIKV E animals for each group. Asterisks indicate significance compared to the PBS control group at day 10 by one-way ANOVA with Dunnett’s multiple comparisons test. **C** Survival of immunized and control animals. Survival curves were compared individually to the PBS control group (colored asterisks) or across groups (black asterisks) using a Mantel–Cox log-rank test. For all analyses, ****P < 0.0001, ***P < 0.001, **P < 0.01, *P < 0.5 and ns: not significant (*P* > 0.5).

To evaluate the immunogenicity of the recombinant ZIKV DI-DIII subunit immunogen and its ability to elicit protective responses in vivo, we used the stringent C57BL/6 type I-interferon receptor knock-out (*Ifnar1*^-/-^) ZIKV challenge mouse model^30,31^. We immunized *Ifnar1*^-/-^ C57BL/6 mice using a homologous prime/boost strategy with either ZIKV DI-DIII, DIII, or full-length E (ZIKV E) as a benchmark at days 1 and 21, followed by a lethal ZIKV challenge at day 55 (Fig. 3A). The protein immunogens were formulated with a combination of alhydrogel and CpG2395 to adjuvant the immune response. Mice were monitored up to 30 days post-challenge for weight and survival, across 3 independent experiments. The saline and adjuvant-only control groups showed significant weight loss reaching nearly 30% at day 10 post-challenge. Only the ZIKV DI-DIII or ZIKV E vaccinated groups were protected from weight loss. The weight curve of the recombinant DIII immunized group mirrored that of the two control groups, with significant weight loss ( > 25%) observed by day 10 (Fig. 3B). Mice that received the ZIKV DI-DIII or ZIKV E were largely protected with >85% of the animals surviving the challenge, with no statistical difference between the two groups (Fig. 3C). In contrast, control mice that received saline, or adjuvant only, succumbed to infection 11 to 14 days post-challenge. The DIII-immunized group showed statistically significant higher mortality, with a majority of animals succumbing to severe disease by day 12 (Fig. 3C).

### Assessment of humoral responses elicited by the ZIKV DI-DIII protein immunogen

**Fig. 4:**
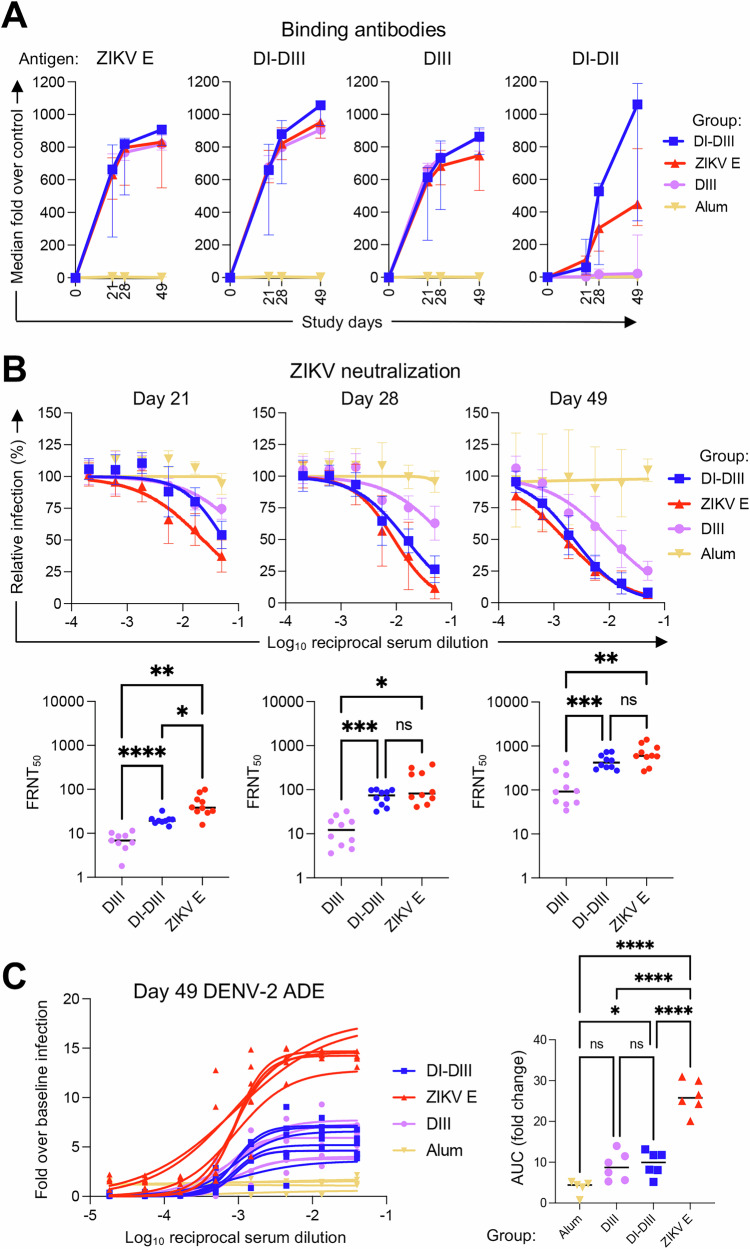
Characterization of binding and neutralizing antibodies in serum of immunized animals. **A** Median ZIKV binding antibody titers across groups with interquartile range, expressed as fold-over a negative serum control, against ZIKV E full-length and subunits, tested in a bead-based multiplex binding assay **B** (Top) ZIKV neutralization curves obtained from a Foci Reduction Neutralization Test (FRNT) against live ZIKV, using 3-fold serum dilutions, starting at 1:20 from 10 animals assessed from two independent animal experiments. Shown are mean relative infection % with standard deviation. (Bottom) Fifty percent inhibitory sera reciprocal dilutions are plotted for each time points across treated groups. Differences between groups were analyzed using Brown-Forsythe and Welch one-way ANOVA with Dunnett’s T3 multiple comparisons test. **C** (Left) Antibody-dependent enhancement (ADE) of DENV-2 infection assay across groups, using four-fold serial dilutions of day 49 serum samples. ADE is expressed as fold over baseline infection. Shown are mean with standard deviation from 6 mice per group analyzed from two independent animal experiments fitted using a 4-parameter non-linear regression analysis. (Right) Differences in area under the curve (AUC) for each mouse between groups were analyzed using one-way ANOVA with Tukey’s multiple comparisons test. For all analyses, ****P < 0.0001, ***P < 0.001, **P < 0.01, *P < 0.5 and ns: not significant (*P* > 0.5).

To assess antibody responses in the serum of immunized animals, we first measured the levels of binding antibodies to a panel of flavivirus antigens. We utilized full-length ZIKV E as well as E subunits to map binding responses to individual domains. Using a custom multiplex bead assay, we observed that by day 21, following the first vaccination, all immunized groups displayed high levels of binding antibodies to ZIKV E, DI-DIII and DIII, that were further boosted following the second immunization (Fig. 4A). Binding antibodies to the ZIKV E DI-DII subunit only became detectable after the second immunization for the animals immunized with the DI-DIII and ZIKV E immunogens, indicating that responses to DI within the DI-DIII construct were likely subdominant after the prime but significantly expanded following the boost. To further map the specificity of peak antibody responses, day 49 sera were depleted of binding antibodies targeting DIII using DIII-displaying yeasts. DIII depletion reduced antigen-specific binding antibodies by two-fold in sera from the ZIKV DI-DIII vaccinated group, indicating that half of the elicited antibodies targeted DI or the DI/DIII interface (Fig. S2A). Next, we developed a DI-DIII mutant harboring 3 amino acid mutations that abolished MZ4 binding to determine whether MZ4-like antibodies had been elicited (Fig. S2B). This mutant DI-DIII protein was able to bind DIII mAbs Z004 and ZKA190, but completely abrogated all binding of MZ4 (Fig. S2C). A modest, but significant drop in binding antibodies was observed from the sera of vaccinated mice at day 49 between wild-type DI-DIII and the mutant protein, indicating the presence of MZ4-like antibodies among the total polyclonal response (Fig. S2D). To further gain evidence for the presence of MZ4-like antibodies, we also performed competition assays using either MZ4 or an HIV monoclonal antibody (VRC01), as a negative control. MZ4 competed against polyclonal antibodies for binding to DI-DIII and the ZIKV E proteins, both harboring the MZ4 epitope, but did not compete for binding to the DI-DIII mutant that did not harbor the MZ4 epitope, as expected (Fig. S2E). MZ4 competed over 30% of the binding responses when pre-incubated with the antigens, confirming that antibodies to the DI/DIII interface represented a significant proportion of the elicited antibodies (Fig. S2E).

Finally, to investigate the ability of these regimens to elicit cross-reactive antibody responses, we used a parallel cohort of mice which were immunized in identical manor but were not challenged, allowing for additional blood collection at a late time point (day 118). Antibody binding cross-reactivity was assessed using E glycoproteins from 7 related flaviviruses, DENV1-4, Japanese encephalitis virus (JEV), Tick-borne encephalitis virus (TBEV) and Yellow fever virus (YFV) using a custom Luminex multiplex bead assay. Detectable, but low levels of cross-reactive antibodies were observed following the boost at days 49 and 118 in all immunized groups, consistent with a primary exposure to ZIKV (Fig. S3). Interestingly, the DIII group elicited notable TBEV cross-binding antibodies with the DI-DIII group eliciting slightly lower responses (Fig. S3). These results suggest that the DIII construct may be more accessible to present cross-reactive epitopes to TBEV compared to the other immunogens, whereas the DI portion could occlude the presentation of these epitopes on DIII that are cross-reactive with TBEV E.

Having observed strong ZIKV binding antibody responses in all vaccinated groups, we next evaluated neutralizing responses in time points leading to challenge. Using a foci reduction neutralization test (FRNT), we observed that the DI-DIII immunogen group exhibited high ZIKV neutralization titers starting at day 21 that further increased with subsequent boosting. This neutralization potency was comparable to the ZIKV E group after 2 vaccinations, with a median titer of 75 at day 28 and over 400 at day 49 post-boost (Fig. 4C, bottom). Neutralization titers for the DIII-treated group were consistently and significantly lower at all time points and only reached a maximum median titer of 90 at day 49; likely explaining the lack of in vivo protection in our high dose challenge model (Fig. 3B–C). To further interrogate the specificity of the neutralizing antibodies elicited by the ZIKV DI-DIII immunogen, we again tested the day 49 DIII-depleted sera for neutralization and observed that approximately 50% of the neutralizing fraction remained following DIII depletion (Fig. S2A, right), suggesting that neutralizing antibodies may target multiple epitopes throughout DI, DIII, and the DI-DIII interface. Having detected weak binding antibody cross-reactivity to other flaviviruses, we also examined the neutralizing potential of serum from vaccinated animals against the four serotypes of DENV at the late day 118 time point, where a complete lack of neutralization was detected to DENV 1-4 (Fig. S4A). These data suggested that mice vaccinated with ZIKV DI-DIII mounted ZIKV-specific neutralizing responses against epitopes both within and outside of DIII, including the DI or the DI/DIII interface.

While the groups immunized with the ZIKV DI-DIII immunogen or ZIKV E displayed similar neutralizing activity following the boost, we next wanted to evaluate the potential for enhancement of dengue infection in vitro. To test this, we evaluated day 49 sera, that showed modest but detectable levels of cross-reactive antibodies, from all immunized groups for their ability to enhance DENV-2 infection in vitro. We observed significantly higher DENV-2 infection following incubation with sera from the ZIKV E group (median AUC fold change of ~25), compared to the other groups (Fig. 4E). A modest ( ~ 9-fold) enhancement was obtained with sera from the recombinant ZIKV DI-DIII and DIII groups compared to the adjuvanted group (4-fold), but no difference was found between these groups (Fig.4E). To further investigate the potential for ADE of other DENV serotypes, we used sera from day 118 to quantify ADE using the same in vitro assay as described for DENV-2. Here, we observed very similar results where immunization with ZIKV E generated antibodies capable of ADE, while the DI-DIII and DIII immunogens had a significant reduction in ADE potential for DENV-1, -3, and -4 (Fig. S4B). Taken together, these data indicate that both the ZIKV DI-DIII and ZIKV E immunogens elicited potent neutralizing and protective responses in mice, but that recombinant ZIKV DI-DIII generated significantly less enhancement of DENV infection in vitro.

## Discussion

In this study, we engineered a ZIKV DI-DIII subunit immunogen by deleting the DII domain of ZIKV E and focusing antibody responses on known viral targets of potent neutralizing antibodies. Structural studies revealed that the recombinant DI-DIII subunit adopts a conformation essentially identical to its native fold within the E protein dimer on the virion surface. Remarkably, mice immunized with DI-DIII developed high titers of neutralizing antibodies and were protected from lethal ZIKV challenge, similar to animals immunized with the full-length E, but with significantly lower ADE, demonstrating DI-DIII as an alternative immunogen to full-length E or virus-like particles presenting full-length E.

Development of a ZIKV vaccine is critical to protect vulnerable populations, both flavivirus -naïve and -experienced. This current study demonstrated that an immunogen consisting of the ZIKV DI-DIII domains retained its original conformation and protected mice from lethal ZIKV challenge when used as a recombinant protein. DIII-depleted sera, antibody competition studies, and testing of the MZ4 binding mutant confirmed that antibody responses were directed towards multiple epitopes presented on DI, DIII, or the DI/DIII interface and may have contributed to the protection observed. Our mouse study indicated that vaccination with DI-DIII, in a flavivirus-naïve context, elicited ZIKV responses with modest levels of cross-reactivity observed against related flaviviruses, as expected from a ZIKV primary exposure^32^. These low-level cross-reactive binding antibodies to DENV 1-4 did not mediate neutralization, and therefore, we hypothesize that these cross-reactive antibodies would not mediate protection against DENV. However, more importantly, we found that DI-DIII may have diminished the potential of ADE of across all DENV 1-4 serotypes in vitro, in contrast to full-length ZIKV E. This result supported our hypothesis that removal of DII, and its highly conserved fusion loop epitope, would reduce the majority of undesirable responses in flavivirus-naïve individuals.

Knowing the diversity of the B cell repertoire following flavivirus infection, we envisage the potential for clinical applications for a ZIKV DI-DIII-based immunogen in flavivirus-experienced participants, where the presence of imprinted cross-reactive memory B cells directed to neutralization epitopes on DI-DIII from prior flavivirus exposure could be boosted for cross-protection. Indeed, MZ4 is a potent, cross-neutralizing antibody against ZIKV and DENV 1-4, suggesting that immunogens designed to recall imprinted B cells targeting this epitope may yield cross-neutralization across these flaviviruses. Conversely, the presence of imprinted cross-reactive memory B cells directed to the FLE in DII originating from prior exposure, could hamper downstream flavivirus vaccine responses. We recently reported that YFV vaccination 6 months prior to ZPIV vaccination, delayed the generation of ZIKV neutralizing responses, compared to a flavivirus-naïve control group^33^, although binding antibodies to the ZIKV E glycoprotein were similar. A 3^rd^ ZIKV immunization was required for the YFV-exposed participants to reach neutralization titers similar to those with only two ZPIV vaccinations in the flavivirus-naïve group^33^. While the exact mechanism(s) of delayed ZIKV neutralization in YFV-primed, ZPIV-vaccinated individuals is being investigated, we hypothesize that immunization with the recombinant ZIKV DI-DIII immunogen in flavivirus-experienced individuals would elicit de novo B cell responses that target ZIKV-specific epitopes on the DI-DIII subunit, which may have resulted in higher neutralization and protective responses. In contrast, a vaccine including the full-length E may primarily recall pre-existing memory B cells targeting poorly neutralizing conserved epitopes in DII, such as FLE, leading to a decrement in neutralization titers following the first immunization series. The same may be true in the case of DENV- or JEV-experienced participants^33^, although unlike the more distantly-related YFV, DENV and JEV serotypes share more cross-neutralizing epitopes with ZIKV (i.e., within DIII^21,23–25^ and the DI/DIII interface^18^), suggesting that immunization with ZIKV DI-DIII, in this context, may also recall memory B cells of neutralizing potential.

In flavivirus-naïve individuals that do not live in flavivirus endemic areas, such as individuals largely here in the US, we envisage the ZIKV DI-DIII to elicit responses that focus antibody responses on neutralization epitopes present on the DI-DIII immunogen. If these ZIKV DI-DIII immunized individuals travel to flavivirus endemic regions, then we hypothesize that the potential of cross-reactive responses that permit ADE would be blunted, and these individuals would be at a decreased risk of developing severe dengue while having protection against ZIKV infection. Overall, the goal of this immunogen is to elicit protective responses to ZIKV by focusing the immune response to neutralization epitopes present on the DI/DIII interface while limiting the potential of ADE. The limitations of this study include studying the potential of ADE in vitro, which may not equate to the potential of ADE in vivo. In addition, all in vivo studies were conducted in mice which may not represent responses observed in humans. Follow up studies will use developed vaccine candidates to assess protection in non-human primates against ZIKV challenge and protection against vertical transmission in utero.

We describe here a recombinant ZIKV DI-DIII immunogen, but we anticipate that DI-DIII immunogens can be readily designed for a variety of related flaviviruses, as recently reported for DENV^34^. Future developments of ZIKV DI-DIII will focus on the use of multivalent display on nanoparticles to further enhance its immunogenicity in combination with potent adjuvants, a platform we successfully used for a SARS-CoV-2 vaccine candidate^35,36^, as well as designing iterative vaccinology approaches to potentially identify ideal combinations for a safe and efficacious ZIKV-DENV protective vaccine. In summary, we have designed a novel ZIKV E immunogen that lacks DII and presents flavivirus neutralizing epitopes, affording equivalent protection from challenge, compared to full-length E, with reduced ADE. These studies provide a proof-of-concept for future development of DI-DIII vaccine platforms aimed at providing protective immunity to ZIKV in populations with or without prior exposure to flaviviruses.

## Methods

### Ethics statement

All animal studies were conducted under an IACUC-approved animal use protocol in an American Association for Accreditation of Laboratory Animal Care (AAALAC) Internationally accredited facility with a Public Health Services Animal Welfare Assurance and in compliance with the Animal Welfare Act and other federal statutes and regulations relating to laboratory animals. This research adhered to the principles stated in the Guide for the Care and Use of Laboratory Animals, NRC Publication, eighth edition, 2011. These studies were approved by the Saint Louis University Animal Care and Use Committee (IACUC; protocol 2771) and the University of Kentucky (IACUC; protocol 2023-4366).

### Cells and viruses

Drosophila S2 and human Expi293F cells were purchased from ThermoFisher and cultured according to the manufacturer’s instructions. Vero-RCB (World Health Organization) were cultured at 37 °C in Dulbecco’s Modified Eagle Medium (DMEM) supplemented with 5% (v/v) fetal bovine serum (FBS), and 10 mM HEPES (pH 7.3). Virus stocks were titered by a focus-forming assay on Vero cells^37^. ADE assays were completed using K562 cells, a human leukemia cell line (kind gift of Dr. Jacki Kornbluth, Saint Louis University).

ZIKV (strain PRVABC59, GenBank: KX087101) was obtained from the CDC and grown on Vero cells. ZIKV was passaged once in Vero cells and titrated by focus-forming assay. DENV-1 (strain West Pac 74, GenBank: PX492104) was obtained from Dr. Carlos Sariol, DENV-2 (strain D2S10, GenBank: JN796245) was obtained from Eva Harris, DENV-3 (strain C0360/94, GenBank: KJ737429) was obtained from Alan Barrett, DENV-4 (strain TVP376, GenBank:KC963424) was obtained from Robert Tesh. All four DENV serotypes were grown on C6/36 (Kind gift of Dr. Greg Ebel, Colorado State University) at 32 °C in Dulbecco’s Modified Eagle Medium (DMEM) supplemented with 5% (v/v) fetal bovine serum (FBS), and 10 mM HEPES (pH 7.3).

### Production of recombinant proteins

All recombinant flavivirus E proteins and domain subunits (Supplementary Table 2), except for ZIKV DIII and the DI-DIII mutant, were produced using the Drosophila Expression System (ThermoFisher), as per the manufacturer’s instructions. Briefly, codon-optimized coding sequences, with C-terminal AviTag and 8x histidine tags, were synthesized by Genscript and cloned into the pMT-BiP vector (ThermoFisher), in-frame with the insect BiP secretion signal and under the control of the inducible Metallothionein (MT) promoter. S2 cells were co-transfected with the respective pMT-BiP expression vector and the pCoBlast selection vector at a 19:1 (w/w) ratio. Stably transfected cell pools were selected with Blasticidin and adapted to suspension culture in serum-free medium (Lonza InsectXpress). Expression was induced with 0.5 mM CuSO4 and culture supernatants were harvested after seven days. The coding sequence for ZIKV DIII domain was synthesized (Genscript) and cloned into the pcDNA3.4 vector (ThermoFisher) downstream from a murine Ig leader sequence. ZIKV DIII and the DI-DIII mutant were produced by transient transfection in Expi293F. All proteins were purified by affinity chromatography on a Ni-NTA resin (Qiagen), followed, when necessary, by gel filtration on an ENrich SEC 650 column (Bio-Rad) to obtain pure ( > 90%) monomeric proteins. Purity and integrity of all recombinant proteins were assessed by SDS-PAGE, Coomassie staining and binding assays with control antibodies. Monoclonal antibodies MZ4^18^, 2A10G6^29^, ZKA190^22^, Z004, Z006^21^ and VRC01^38^ were produced recombinantly as human IgG1 from the available public sequences by transient transfection in Expi293F cells and Protein A affinity chromatography. MZ4 Fab was produced by IgG1 digestion with endoproteinase LysC (New England Biolabs) at a ratio of 1:2000 (w/w), for 3–5 h in a shaker incubator at 37 °C. Digestion was assessed by SDS–PAGE and, upon completion, the reaction mixture was passed through Protein A resin (Cytiva) three times and the final flowthrough was assessed by SDS–PAGE for purity.

### Biolayer interferometry

Real-time interactions between purified ZIKV DI-DIII and antibodies were monitored on an Octet RED96 instrument (FortéBio). Recombinant ZIKV DI-DIII was biotinylated with the BirA biotinylation kit (Avidity), diluted in kinetics buffer (0.1% (w/v) bovine serum albumin (BSA), 0.02% (v/v) Tween-20 in PBS; FortéBio) and immobilized on streptavidin (SA) biosensors (FortéBio), at ~50% of the sensor maximum capacity. Baseline was established in kinetics buffer. To evaluate binding of control antibodies, biotinylated ZIKV DI-DIII loaded SA biosensors were dipped into wells containing mAbs (IgG1) at 400 nM for an association step of 450 s, followed by a 1000 s dissociation step in kinetics buffer. To measure binding affinities, biotinylated ZIKV DI-DIII loaded SA biosensors were dipped into wells containing serial dilutions of MZ4 Fab (from 12.5 to 1.5 nM) for 400 s. Complexes were then allowed to dissociate in kinetics buffer. After reference subtraction, apparent binding kinetic constants were determined from at least four concentrations of Fab by fitting the curves to a 1:1 binding model using the Data Analysis software 9.0 (FortéBio).

### X-ray crystallography and structural analysis

The complex of ZIKV E protein DI-DIII with MZ4 Fab at ~10 mg/mL was screened for crystallization conditions using an Art Robbins Gryphon, 0.4 µL droplets, and a set of ~1000 conditions. Crystals for data collection were grown in MIDASplus screen (Molecular Dimensions) condition C6- 40% (v/v) pentaerythritol propoxylate (5/4 PO/OH) and 15% (v/v) ethanol. Crystals were cryoprotected with addition of glycerol to 20% (v/v). Diffraction data were collected at the FMX beamline at the National Synchrotron Light Source-II (Brookhaven National Lab). Data were indexed, integrated, and scaled utilizing autoPROC^39^, as implemented by the FMX data processing pipeline. Phenix xtriage^40,41^ was used to analyze all scaled diffraction data output from autoPROC for measurement value significance, completeness, asymmetric unit volume, and possible twinning or pseudotranslational pathologies. The ZIKV DI-DIII with MZ4 Fab complex structure was determined by molecular replacement with PHASER^42^ in Phenix. The ZIKV E-MZ4 complex (PDB ID 6NIU) was edited to remove DII, CDRs, and all flexible loops; this revised model was the search model used for MR. The structure was iteratively built with Autobuild in Phenix^43^ and manually in Coot^44^ followed by refinement with Phenix Refine^45^. Model quality was assessed with MolProbity^46^. MZ4 contact residues were identified using PISA (https://www.ebi.ac.uk/pdbe/pisa/). Data collection and refinement statistics are reported in Table S1. Structure figures were generated using PyMOL (version 2.5.2; Schrodinger, LLC) and UCSF Chimera^47^ package (version 1.19) from the Resource for Biocomputing, Visualization, and Informatics at the University of California, San Francisco (supported by NIH P41 RR001081). R.M.S.D. values were calculated for the structures of MZ4 in complex with full-length E vs DI-DIII (Fig. 2A). R.M.S.D. values for the MZ4 heavy and light chains were determined via alignment of the heavy or light chains of the MZ4-DI-DIII structure with the heavy or light chains of the MZ4-ZIKV E structure. All R.M.S.D. calculations were performed in PyMOL (version 2.5.2).

### Animal infections and treatments

C57BL/6 and *Ifnar1*^*-/-*^ mice homozygous for interferon alpha and beta receptor subunit knockout mutation on the C57BL/6 J background (B6.129S2-Ifnar1^tm1Agt^/Mm, Strain #:032045) mice were ordered from The Jackson Laboratory. Mice were bred in a pathogen-free mouse facility at the Saint Louis University School of Medicine and the University of Kentucky where experiments were performed in accordance with and approval of Federal and University regulations. For in vivo ZIKV studies, five, six-week-old mice were immunized with 5 μg intramuscularly (IM) on days 1 and 21. All vaccinations and subsequent virus inoculations were performed under anesthesia, ketamine/xylazine cocktail, administered intraperitoneally (IP). Mice were challenged by intravenously (IV) injection of 100 μL of virus (1 × 10^5^ FFU/mouse), which was a dose and route optimized to allow viral replication and measurable severe disease. Mice were anesthetized during this procedure using Ketamine/Xylazine (90 mg/kg: 10 mg/kg). After challenge, each mouse was examined for visible trauma and placed back into its cage for recovery. A parallel cohort of mice (*n* = 5 per treated group) received the same vaccination regimen but were not challenged at day 55, allowing for an additional blood collection at day 118, when the animals were humanely euthanized.

### Antibody binding multiplex assay

A panel of 12 flavivirus antigens with C-terminal Avi-tag were biotinylated at a 1:1 molar ratio using the BirA500 kit (Avidity) overnight at 4 °C. Excess biotin was removed via size exclusion over a Zeba column (ThermoFisher). Streptavidin (Agilent) was coupled to uniquely coded carboxylated magnetic microspheres (Luminex Corp) per manufacturer’s protocol. Streptavidin-coated microspheres were incubated with biotinylated antigens at a 1:4 molar ratio for 1 h at 4 °C on an end-over-end rotor, washed to remove unbound protein and then incubate with free biotin for 30 min to block any unbound streptavidin binding sites. Antibody binding and characterization was performed as previously described^48–50^, with minor modifications. Mouse sera diluted 1:100 in assay buffer were incubated with pooled antigens for 2 h at room temperature, washed then incubated for 1 h with phycoerythrin labeled goat anti-mouse IgG (Southern Biotech) at 1 µg/mL. After a final wash to remove unbound detection reagent, microspheres were resuspended in 40 μL sheath fluid (Luminex Corp) and data were collected on a Bio-Plex^®^3D Suspension Array system (Bio-Rad, Hercules CA) running xPONENT^®^ v.4.2 (Luminex Corp). The median signal from the pre-study visits of all animals served as the negative control and data were analyzed as fold over the negative control. The median signal was reported for each immunized group of mice. To assess differences in binding between ZIKV DI-DIII WT and mutant, median signal were reported from the sera of all groups of immunized mice. A pan-flavivirus mouse serum positive control was also included. It was generated by infecting mice with a sublethal dose of ZIKV followed by a YFV challenge, 30 days later^51^. The assay was also adapted to assess competition by the MZ4 human monoclonal antibody. For this, the ZIKV DI-DIII, DI-DIII mutant, and ZIKV E bead-bound proteins were pre-incubated with MZ4 or a negative control human antibody (HIV mAb VRC01^38^) at 20 µg/ml overnight prior to incubation with day 49 mouse sera. Bound mouse antibodies were detected using a human IgG-adsorbed phycoerythrin labeled goat anti-mouse IgG, as described above. MZ4 inhibition of polyclonal binding was compared to the negative control for each protein.

### Focus reduction neutralization test (FRNT)

ZIKV: Serum from immunized mice was serially diluted and incubated with 100 FFU of ZIKV for 1 h at 37 °C before infecting a monolayer of Vero WHO cells in a 96-well plate. One hour later, cells were overlaid with 1% (w/v) methylcellulose diluted in 2% (v/v) FBS DMEM. After 30 hours of infection, cells were fixed for 10 min with 5% (v/v) paraformaldehyde and washed twice with PBS. Cells were permeabilized with PermWash buffer (0.1% [v/v] saponin, 0.1% [w/v] BSA in PBS) and stained overnight with m4G2 primary antibody. Cells were permeabilized and stained for 2 h with goat anti-mouse-HRP secondary antibody. Wells were treated with TrueBlue peroxidase substrate to develop focus-forming units, which were then quantified using an ImmunoSpot ELISpot plate scanner. DENV 1-4: An identical experimental approach was used to quantify the neutralization potential of serum from vaccinated mice against DENV serotypes 1-4 with the following differences, serially diluted mouse serum was incubated with either 80 FFU of DENV-1, -2, -3, or -4. After infection and overlaying with methylcellulose of the Vero-WHO cells infection was allowed to proceed for 72 h where then cells were fixed, stained and quantified as described above. A positive mouse serum control was used for each DENV serotype. It was generated by giving Ifnar1^−/−^ mice a sublethal infection with the homologous DENV serotype. The mice were sacrificed 60 days post infection.

### Depletion of ZIKV DIII-reactive antibodies in serum

The DNA fragments encoding amino acid residues 294 to 409 (DIII) of ZIKV E protein were cloned as downstream fusions to Aga2 in the yeast surface display vector pETCON, under the control of an upstream GAL1 promoter. The original plasmid pETcon_SARS-CoV-2_RBD was a gift from Jesse Bloom (Addgene plasmid # 166782; http://n2t.net/addgene:166782; RRID: Addgene_166782). These constructs were transformed into Saccharomyces cerevisiae strain EBY100 to generate yeast that expressed ZIKV DIII. Individual yeast colonies were grown to logarithmic phase at 30 °C in tryptophan-free yeast media containing 2% glucose. Fusion protein expression was induced on the surface by growing yeast for additional 48 h in tryptophan-free media containing 2% (w/v) galactose at 20 °C. Induction of DIII antigen was verified by flow cytometry using the Z006 mAb^21^, followed by goat anti-human IgG secondary antibody conjugated to Alexa Fluor 647 (Biolegend). After verification, yeast was treated with high dose amphotericin (1μg/ml) for 6 h then washed twice with sterile PBS, to allow testing of serum in functional virus neutralization assays. For antigen specific depletion, mouse serum was diluted 1:30 then incubated with a vortexed pellet equal to 1 OD_600_ of ZIKV DIII yeast and incubated overnight at 4 °C in a rotating shaker. Antibody neutralization was quantified in the focus forming assay method at a final serum dilution of 1:60 as described above. We quantified pre- and post-DIII-depletion antibody levels by measuring polyclonal serum binding to yeast displaying ZIKV antigens (DIII, DI–DIII, or E (1-404), matched to the vaccine immunogens. Similar to above, the pETCON yeast display vector was induced to express ZIKV DIII or the soluble domain of E, where expression was measured by anti-myc (rabbit anti-myc–A488, Biolegend) and proper folding measured by Z006 anti-DIII followed by goat anti-human Ig (H + L)-A647 (Biolegend). Serum was diluted 1:30 in sterile PBS and incubated with antigen-displaying yeast. Because induction yields a variable fraction of non-displaying cells, binding was normalized to the fraction of myc-positive events, with the maximum myc signal defined as 100% protein display. Flow cytometry gates were applied sequentially: FSC vs SSC (cells) → singlets → myc-positive yeast → primary serum binding detected with goat anti-mouse Ig (H + L)–A647 (Biolegend). The percent of myc^+^ cells which stain positive with mouse polyclonal sera are represented as percent antibody positive. Antibody bound yeasts were quantified using an Attune NxT flow cytometer (Thermo Fisher).

### Antibody dependent enhancement assay (ADE)

DENV serotypes 1-4 ADE assays were performed and analyzed using procedures previously described to quantify the antibody-mediated enhancement potential of polyclonal antibody immunoglobulin specific for ZIKV^52^ against DENV serotypes 1-4. Four-fold serial dilutions of serum were mixed with either DENV-1 strain West Pac 74, DENV-2 strain D2S10, DENV-3 strain C0360/94 or DENV-4 strain TVP376 at a multiplicity of infection (MOI) of 1 then incubated at 37 °C for 1 h, to allow the formation of immune complexes. Immune complexes were then added to K562 cells that express the Fc-gamma receptor (FcγR) CD32A and incubated for 2 days. Cells were fixed, washed, permeabilized with saponin, and stained with flavivirus cross-reactive mAb rabbit 4G2 antibody at 4μg/ml. After incubating cells for 1 h at 4 °C, cells were washed and stained with A647-conjugated goat anti-rabbit IgG secondary antibody (1:5000), washed, and analyzed on an Attune flow cytometer. The percentage of infected cells was determined using FlowJo 10.4.2 software (Tree Star). ADE was calculated as [% infected cells in the presence of antibody or serum] / [% infected cells in of virus only (no antibody or serum)].

### Statistical analysis

For the animal studies, weight loss in treated groups were compared to the PBS control group by one-way ANOVA with Dunnett’s multiple comparisons test. Survival curves were compared individually to the PBS control group using a Mantel–Cox log-rank test. For neutralization assays, differences between groups were analyzed using Brown-Forsythe and Welch one-way ANOVA with Dunnett’s T3 multiple comparisons test. In the ADE assay, differences in area under the curve (AUC) between groups were analyzed using one-way ANOVA with Tukey’s multiple comparisons test. Wilcoxon paired analysis was used to assess the difference in binding responses observed across antigens and after competition with MZ4. For all analyses, ****P < 0.0001, ***P < 0.001, **P < 0.01, *P < 0.5 and ns: not significant (*P* > 0.5). All tests were performed in Prism (version 10, GraphPad Software). Data were graphed using Prism software (version 10, GraphPad Software).

## Supplementary information

Supplementary Figures

## Data Availability

Data for crystallographic complexes are available from the PDB under accession code 9XYB. Source data are provided within this paper. All other data are available from the corresponding authors upon request.
