# Supplementary material for: Targeting the Zika virus envelope domains I and III as a recombinant vaccine protects mice from lethal challenge
Source: NPJ Vaccines. 2026 Apr 14;11:118. doi: 10.1038/s41541-026-01442-8 (PMC13270120; doi:10.1038/s41541-026-01442-8)
Supplement: Supplementary file 1 — Supplementary Figures [file 41541_2026_1442_MOESM1_ESM.pdf]

Supplementary Figure 1

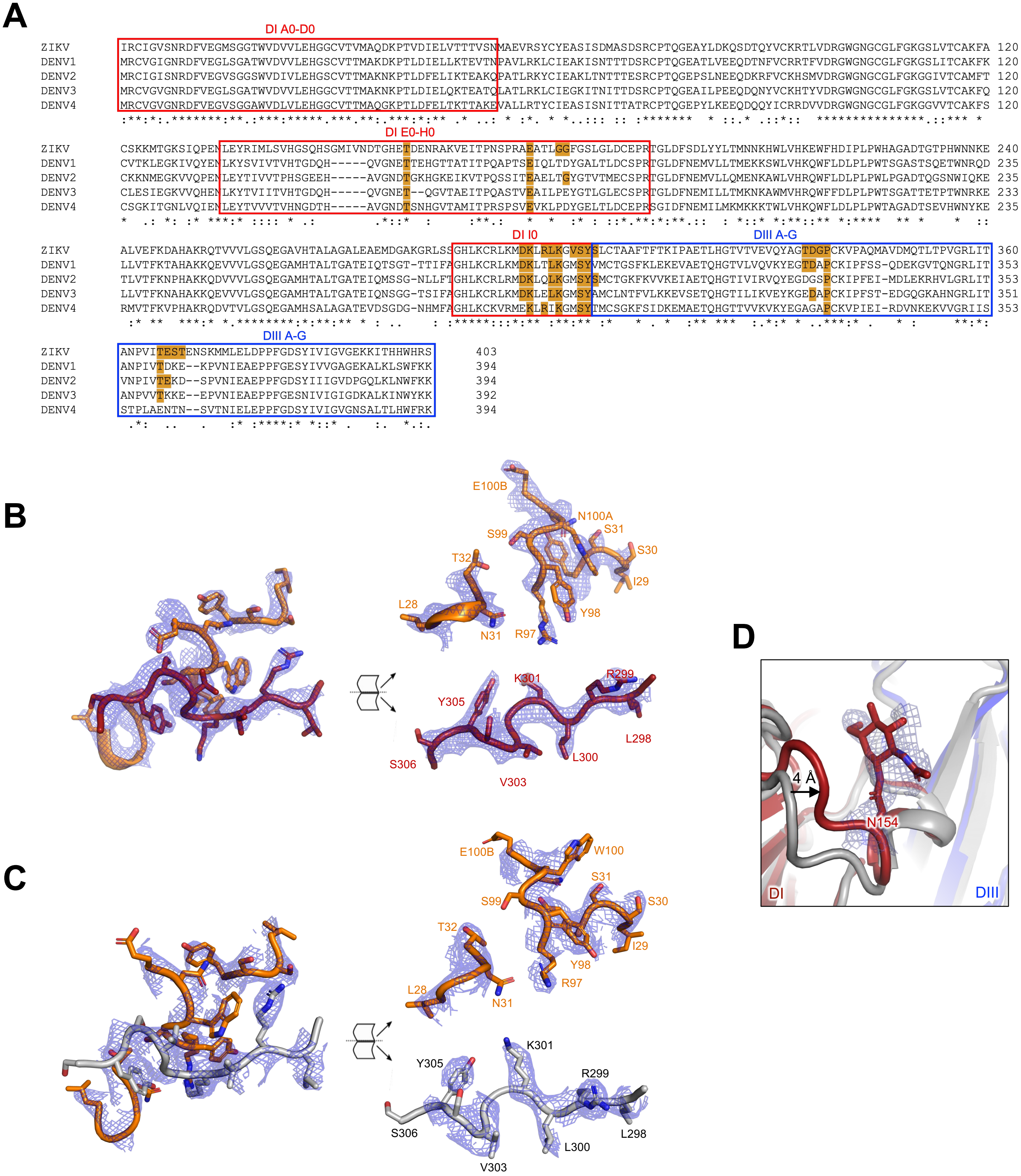

Supplementary Figure 1. Epitope recognition by MZ4. **A**, Sequence alignment of ZIKV, DENV-1, DENV-2, DENV-3, and DENV-4 E proteins. The DI and DIII are boxed in red and blue, respectively, and residues recognized by MZ4 are highlighted in orange. **B**, $2F_o$-F_c electron density for residues at the DI-DIII linker-MZ4 interface of the DI-DIII-MZ4 structure determined to a resolution of 2.9 Å, or **C**, the ZIKV E-MZ4 structure, determined to a resolution of 4.3 Å. Blue mesh is contoured at 1.5σ. **D**, $2F_o$-F_c electron density for the NAG and N154; the 4 Å shift in the DI-DIII structure toward DIII is specified.

Supplementary Figure 2

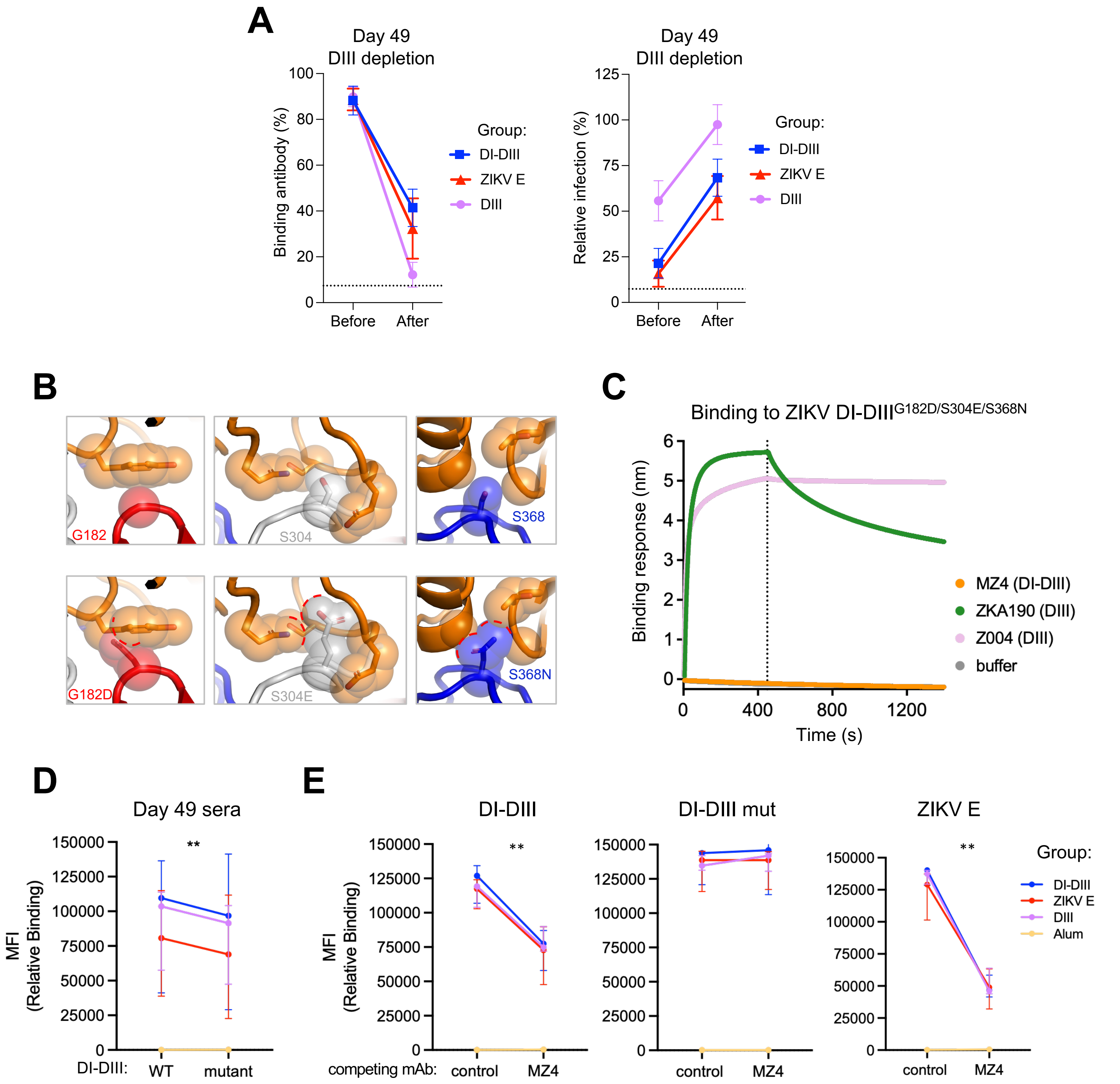

Supplementary Figure 2. Mapping antibody responses in mouse sera. **A**, (Left) Normalized mean binding antibody at 1:30 serum dilution recovered after day 49 serum depletion with DIII-expressing yeasts with standard deviation, in each of the three vaccine arms. (Right) Mean ZIKV neutralization of DIII-depleted day 49 serum at serum dilution 1:60 with standard deviation, for each of the three vaccine arms. Shown are the responses from 5 animals for each group. **B**, (Upper panels) Wild-type (WT) residues at the DI-DIII/MZ4 interface; (lower panels) mutated residues at the DI-DIII/MZ4 binding interface. Likely steric or polar clashes are illustrated with red dashed lines. **C**, Binding characteristics of a ZIKV DI-DIII mutant protein mutated in the MZ4 epitope, as assessed by BLI in the same conditions used for Figure 1D. **D**, Day 49 relative median binding antibody titers with interquartile range against ZIKV DI-DIII wild-type (WT) and mutant (mut) proteins, tested in a bead-based multiplex binding assay. **E**, Day 49 relative median binding antibody titers to ZIKV DI-DIII, DI-DIII mutant, and ZIKV E with interquartile range, as measured in a competition bead-based multiplex binding assay with the beads pre-incubated with the indicated human antibodies (negative control HIV mAb VRC01 or MZ4). (D-E), Shown are the median responses with interquartile ranges noted for 5 animals per each group. Significant decrease in antibody titers was determined using Wilcoxon paired analysis **P<0.01.

Supplementary Figure 3

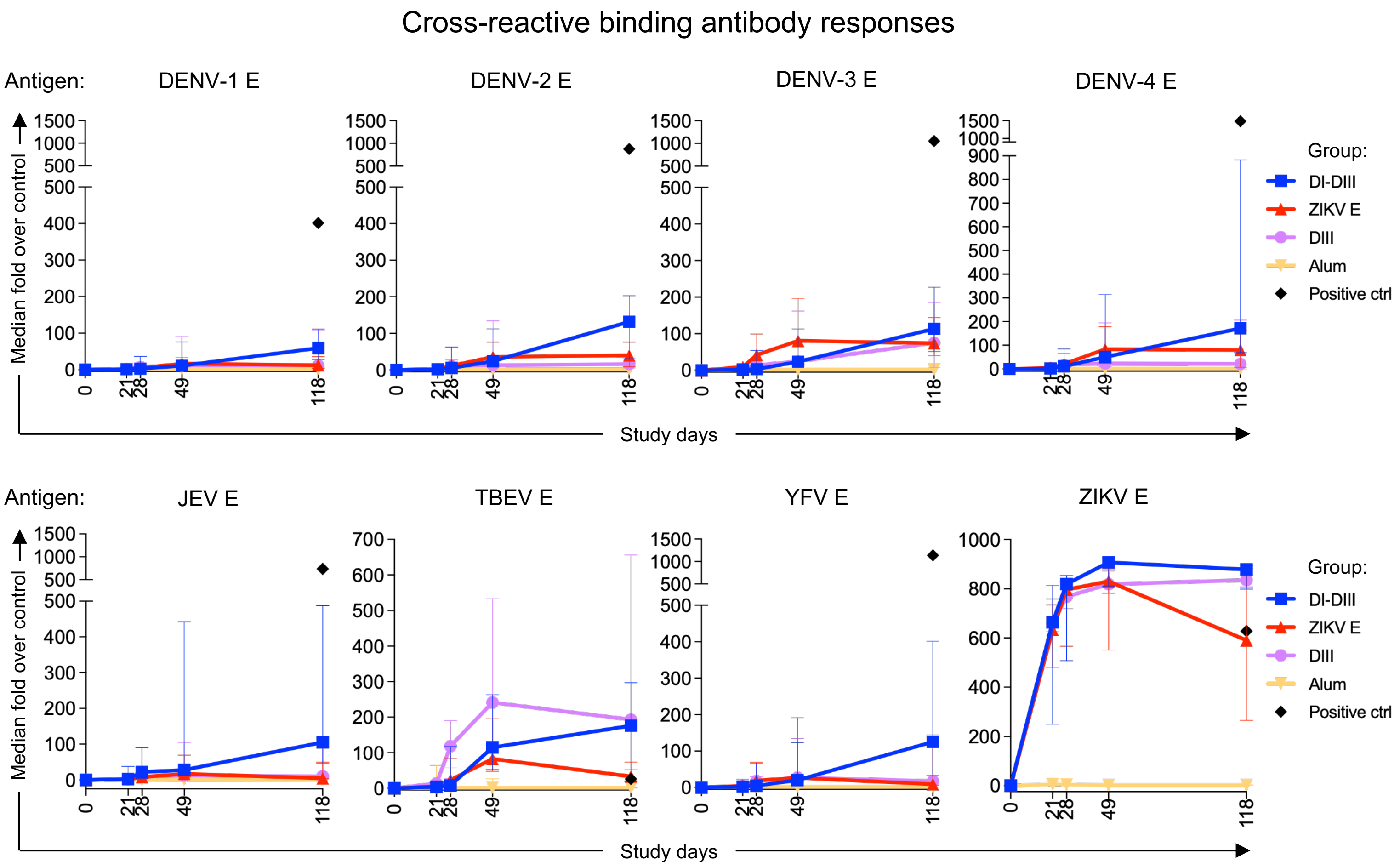

Supplementary Figure 3. Cross-reactive binding antibody responses. Median ZIKV binding antibody titers against the indicated flavivirus E full-length proteins from each group as tested in a bead-based multiplex binding assay. Data is expressed as the median fold-change in response from baseline for 5 mice in each group; errors bars represent the interquartile range. Black diamond indicates the titer obtained with a positive control mouse sera (ZIKV/YFV sequential infection) and is shown at day 118 for reference.

Supplementary Figure 4

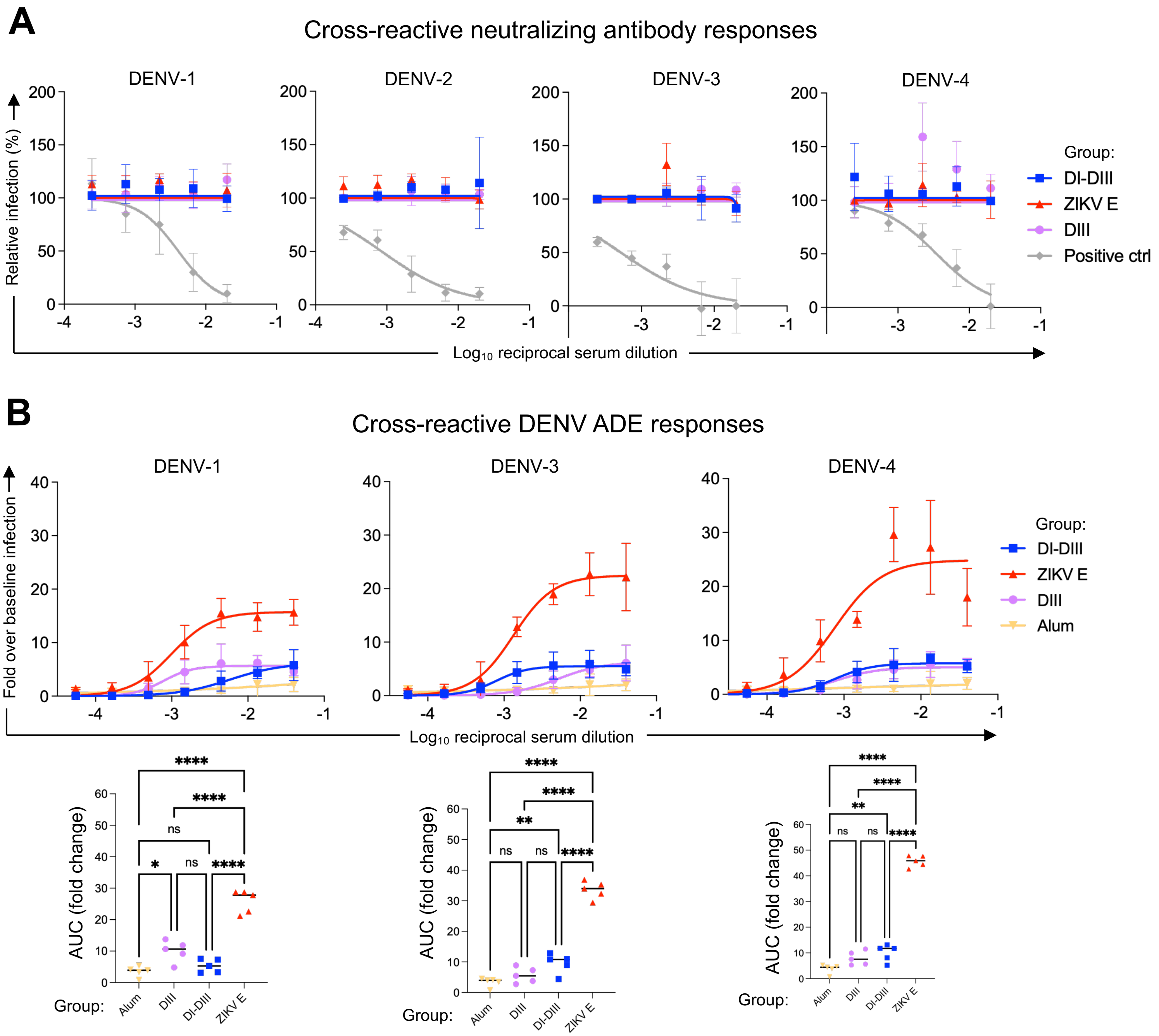

Supplementary Figure 4. DENV neutralizing and ADE responses at day 118. A, DENV neutralization curves obtained from a Foci Reduction Neutralization Test (FRNT) against live virus, using 3-fold serum dilutions, starting at 1:50. Shown are mean relative infection % with standard deviation. A serotype-specific mouse serum positive control was added to each assay (grey diamond). **B**, (Top) Antibody-dependent enhancement (ADE) of DENV infection assays across groups, using four-fold serial dilutions of day 118 serum samples. ADE is expressed as fold over baseline infection. Shown are mean with standard deviation from 5 mice per group in two independent animal experiments fitted using a 4-parameter non-linear regression analysis. (Bottom) Differences in area under the curve (AUC) for each mouse between groups were analyzed using one-way ANOVA with Tukey's multiple comparisons test. For all analyses, ****P<0.0001, ***P<0.001, **P<0.01, *P<0.5 and ns: not significant (P>0.5).

Supplementary Table 1

	ZIKV E DI-DIII with MZ4 Fab (PDB 9XYB)
**Data collection**	
Beamline	NSLS-II FMX
Space group	$P2_12_12$
Cell dimensions	
a, b, c (Å)	135.6, 57.3, 88.1
α, β, γ (°)	90, 90, 90
Resolution (Å)	33.82 – 2.85 (2.90 – 2.85)
R_{meas}	0.334 (2.957)
$I / \sigma I$	3.7 (0.8)
Reflections (tot/uni)	112299 / 16589
Completeness (%)	100 (100)
Redundancy	6.8 (7.2)
$CC_{1/2}$	0.985 (0.421)
R_{pim}	0.127 (1.091)
Wilson B-factor (Å^2)	52.5
**Refinement**	
Resolution (Å)	32.9 – 2.85 (3.03 – 2.85)
No. reflections	16550 (2671)
R_{work} / R_{free} (%)	24.2/31.7
No. atoms	
Protein/ligand/water	4922/14/1
Ramachandran (%)	
Favored/allowed/outliers	96.7/3.3/0.0
B-factors (Å^2)	
Protein/ligand/water	77.4/113.6/99.2
R.m.s. deviations	
Bond lengths (Å)	0.011
Bond angles (°)	1.4

Supplementary Table 1. X-ray data collection and refinement statistics. Values in parentheses pertain to highest resolution shell.

Supplementary Table 2

Protein	# residues	Strain, GenBank #	Expression system	Purification
ZIKV E	1-404	PRVABC59, KX087101	Drosophila S2 cells	Ni-NTA, SEC
ZIKV DI-DII	1-302	PRVABC59, KX087101	Drosophila S2 cells	Ni-NTA, SEC
ZIKV DI-DIII	1-52; 135-196; 286-404	PRVABC59, KX087101	Drosophila S2 cells	Ni-NTA, SEC
ZIKV DI-DIII G182D/S304E/S368N	1-52; 135-196; 286-404	PRVABC59, KX087101	Expi293F cells	Ni-NTA
ZIKV DIII	303-404	PRVABC59, KX087101	Expi293F cells	Ni-NTA
DENV-1 E	1-396	Djibouti, AAK60418	Drosophila S2 cells	Ni-NTA
DENV-2 E	1-396	New Guinea, AAC59274	Drosophila S2 cells	Ni-NTA
DENV-3 E	1-394	Thailand, UCQ65256	Drosophila S2 cells	Ni-NTA
DENV-4 E	1-396	Malaysia, UXX63133	Drosophila S2 cells	Ni-NTA
JEV E	1-401	SA14-14-2, AAK11279	Drosophila S2 cells	Ni-NTA
TBEV E	1-397	NC_001672	Drosophila S2 cells	Ni-NTA, SEC
YFV E	1-394	17D, AFQ32465	Drosophila S2 cells	Ni-NTA

Supplementary Table 2. List of recombinant proteins produced for the study. Purified recombinant full-length E proteins and subunits are indicated with their amino acid residue numbers, viral strain and GenBank accession number, expression host and purification methods.
